# Compliance, persistence, costs and quality of life in young patients treated with antipsychotic drugs: results from the COMETA study

**DOI:** 10.1186/1471-244X-13-98

**Published:** 2013-03-22

**Authors:** Paolo A Cortesi, Claudio Mencacci, Ferrannini Luigi, Elvezio Pirfo, Patrizia Berto, Miriam CJM Sturkenboom, Fabiana L Lopes, Maria G Giustra, Lorenzo G Mantovani, Luciana Scalone

**Affiliations:** 1 Research Centre on Public Health (CeSP), Univeristy of Milan - Bicocca, Monza, Italy; 2 CHARTA Foundation, Milan, Italy; 3 Mental Health Department, Fatebenefratelli Hospital, Milan, Italy; 4 Departement of Mental Health, ASL 3 Genovese, Genoa, Italy; 5 Mental Health Department G. Maccacaro, Turin, Italy; 6 PBE consulting, Verona, and School of Pharmacy, University of Padua, Padua, Italy; 7 Department of Epidemiology & Biostatistics and Medical Informatics, Erasmus University Medical Center, Rotterdam, the Netherlands; 8 Medical Affairs, Janssen-Cilag SpA, Cologno Monzese, Milan, Italy; 9 Dipartimento di Medicina Clinica e Chirurgia, Federico II University, Naples, Italy

**Keywords:** Schizophrenia, Medication compliance, Persistence, Cost of illness, Quality of life

## Abstract

**Background:**

Little data is available on the real-world socio-economic burden and outcomes in schizophrenia. This study aimed to assess persistence, compliance, costs and Health-Related Quality-of-Life (HRQoL) in young patients undergoing antipsychotic treatment according to clinical practice.

**Methods:**

A naturalistic, longitudinal, multicentre cohort study was conducted: we involved 637 patients aged 18–40 years, with schizophrenia or schizophreniform disorder diagnosed ≤10 years before, enrolled in 86 Italian Mental Health Centres and followed-up for 1 year. Comparisons were conducted between naïve (i.e., patients visiting the centre for the first time and starting a new treatment regimen) and non naïve patients.

**Results:**

At enrolment, 84% of patients were taking atypical drugs, 3.7% typical, 10% a combination of the two classes, and 2% were untreated. During follow-up, 23% of patients switched at least once to a different class of treatment, a combination or no treatment. The mean Drug-Attitude-Inventory score was 43.4, with 94.3% of the patients considered compliant by the clinicians. On average, medical costs at baseline were 390.93€/patient-month, mostly for drug treatment (29.5%), psychotherapy (29.2%), and hospitalizations (27.1%). Patients and caregivers lost 3.5 days/patient-month of productivity. During follow-up, attitude toward treatment remained fairly similar, medical costs were generally stable, while productivity, clinical statusand HRQoL significantly improved. While no significantly different overall direct costs trends were found between naïve and non naïve patients, naïve patients showed generally a significant mean higher improvement of clinical outcomes, HRQoL and indirect costs, compared to the others.

**Conclusions:**

Our results suggest how tailoring the treatment strategy according to the complex and specific patient needs make it possible to achieve benefits and to allocate more efficiently resources. This study can also provide information on the most relevant items to be considered when conducting cost-effectiveness studies comparing specific alternatives for the treatment of target patients.

## Background

According to the World Health Organization, there are 24 million people suffering from schizophrenia worldwide, with an average prevalence of around 7 per 1000 adults, mostly in the age group 15–35 years [1]. Schizophrenia is a chronic condition characterized by an early onset, usually during adolescence, severe symptomatology and chronic course [2]. This disease is included among the top ten causes worldwide of long-term and devastating disability, with wide-ranging and long-lasting impact for people suffering from the illness, their families and society as a whole [2-6]. Patients’ clinical condition and wellbeing deteriorate during the course of the disease, with enormous repercussions on their ability to perform daily activities, to work, to have an effective family and social life, and hence on their wellbeing [4-6].

A number of studies have been conducted to estimate different cost items attributable to schizophrenia and its management [6-12]. According to a systematic review of cost of illness studies conducted in the US and several European countries, schizophrenia involves a high magnitude of burden to patients, their families and society: the expenditure for schizophrenia ranges from 1.5 to 3.0% of annual healthcare budget in developed countries [6,10]. Generally, one to two thirds of the total health care cost is related to hospitalizations [7,10-12]. For instance, in the US it has been estimated that schizophrenic patients occupy about 25% of all psychiatric hospital beds, and 40% of all days staying in these hospitals is for schizophrenia-related reasons [11]. In the UK, the use of hospital inpatient care by people with schizophrenia is substantial: in 2006–2007, 34,407 admissions were reported for schizophrenia and related disorders in England, resulting in 2,232,724 inpatient bed days. This amounted to 16% of all admissions and 34% of all bed days related to psychiatric inpatient care [12]. Among the total direct costs, hospitalizations can account for up to 94% [6], while drugs generate lower costs (between 2 and 13%) [6,13,14]. A considerable contribution to the cost to society comes from the indirect costs (i.e., productivity losses), because of employment difficulties, high early mortality rate and patients’ families loss of productivity. According to some authors, schizophrenia-related indirect costs account for approximately half of the total cost of illness [6,9,10,13-15]. In the United Kingdom, it has been estimated that indirect costs are four times higher than direct costs [16]. Other significant cost items have also been identified, like those involving the criminal justice system and related to the impairment of wellbeing (intangible costs), although these costs are difficult to estimate [6].

Some research has shown that compliance to treatment is associated with improvement in Health Related Quality of Life (HRQoL) [17,18] and clinical outcomes [19-21]. Other studies show that outcomes in patients with a first-episode psychosis may be improved by early intervention treatment and reduction of the untreated period [22-27], but long term effects are still not clear [26-28].

Despite the several studies available on some aspects related to the burden of disease, a complete picture of the real-world societal costs and treatment outcomes is still lacking, especially on young schizophrenia patients.

The aim of this study was to assess compliance and attitude toward antipsychotic drug treatment, persistence, clinical status and HRQoL, and to estimate health care costs and loss of productivity in young adult patients diagnosed with schizophrenia or schizophreniform disorder, who needed long term antipsychotic treatment.

## Methods

### Subjects and procedures

We conducted a naturalistic, longitudinal, ambispective (i.e., both retrospective and prospective) multicentre cohort study, named COMETA (*COMpliance, costi e qualità della vita. Esperienze cliniche nella Terapia con Antipsicotici*).

Patients were consecutively enrolled during 2006 and 2007 in 86 Mental Health Centres throughout Italy. To be considered eligible, patients had to satisfy the following inclusion criteria: age from 18 to 40 years, diagnosis of Schizophrenia or Schizophreniform disorder according to the DSM-IV criteria [29], illness duration of 10 years or less before study entry, necessity of long term antipsychotic treatment. At enrolment, the patients had to be in treatment with oral antipsychotic drugs, either started previously or started at the time of enrolment. Among the eligible patients, if they were visiting the centre for the first time and starting a new treatment regimen (i.e., starting a new drug or a new dosage), these patients were classified as “*naïve*”.

Patients were not eligible if they had concomitant diseases for which there was life expectancy of less than 52 weeks, if at the time of enrolment they were hospitalized or staying in Residential Care Units, if they were attending a specialist centre for the care and treatment of people addicted to drugs or alcohol, or if they were pregnant or breast-feeding.

At the enrolment examination, patients’ data attributable to the previous 90 days were collected (retrospective period) and they were then targeted to be followed up for up to 12 months (prospective period). During the prospective follow-up, data collection occurred at 12 ± 2 (*follow-up examination 1*), 36 ± 2 (*follow-up examination 2*), 52 ± 2 (*follow-up examination 3*) weeks: a 2-week time variation per examination was allowed in order to make the occurrence of examinations coincident with those established according to clinical practice.

The study was performed in accordance with the guidelines of the International Conference on Harmonization for Good Clinical Practice as stipulated in the Declaration of Helsinki [30]. Local Ethics Committees approval was obtained at each of the participating study sites. To participate, each patient had to sign an informed consent form.

### Assessments

In order to achieve good quality of the data collected and a good level of inter-rater reliability, the centres participated in two kick-off meetings before the study started, within a 3-years long educational program in which the raters (clinical investigators) who collected the data and assisted the patients during data collection were trained for the completion of all the instruments used in the project.

Data on socio-demographic characteristics, clinical status, HRQoL, drug treatment, compliance and attitude toward antipsychotic treatment, use of health care resources, and loss of productivity were collected by means of case report forms, scales/questionnaires completed by the physician (socio-demographic and clinical data, costs, opinion on patients’ compliance) or by the patient (HRQoL and attitude toward antipsychotic treatment).

*Socio-demographic data* included patients’ gender and age, education, family and working status, and receipt of economic support for their condition.

*Clinical data* were based on diagnosis (schizophrenia vs. schizophreniform disorder), disease severity, type of schizophrenia, duration of disease, age at onset of symptoms, first treatment, first hospitalization, psychiatric symptoms. Functioning and disease severity were assessed using the Positive And Negative Syndrome Scale (PANSS), the Clinical Global Impression – Severity (CGI-S) and the Global Assessment of Functioning (GAF) scales. PANSS is a 30-item scale measuring the presence of positive and negative syndromes in schizophrenia patients and the severity of psychopathology in the week before the day of assessment. Higher scores indicate more severe psychopathology [31,32]. CGI-S assesses clinical symptom severity in the week before the day of assessment, with a score ranging from 1 (not ill) to 7 (among the most severely ill patients) [29,33,34]. With the GAF scale we measured the overall level of symptomatology and social functioning during the previous 3 months, on a scale from 0 to 100, with higher values indicating higher levels of functioning [29,35].

In order to assess their *HRQoL*, patients completed the EQ-5D [36] and the SF-36 (Short-Form 36-item Health Survey) [37]. These questionnaires were chosen for their ability to evaluate both the physical and psychological component of HRQoL and both have proved to be suitable for self-administration in patients with schizophrenia [38-41]. With EQ-5D, the respondents are asked about their HRQoL on the current day. It consists of two main parts: the first part generates a health profile (EQ-5D profile) consisting of 5 domains, namely “mobility”, “self care”, “anxiety or depression”, “usual activities” and “pain or discomfort”, each with three levels of severity (“no problem”, “some/moderate problems”, “extreme problems/impossible to do”). The second part of the questionnaire consists of a visual analogue scale (EQ-5D VAS), measuring overall HRQoL ranging from 0 (worst imaginable health state) to 100 (best imaginable health state). Results from the EQ-5D descriptive system were converted into a utility score by means of an algorithm that uses population-based (social) values. Because specific conversion values for the Italian population are not yet available, we used social values from the United Kingdom in order to convert our EQ-5D descriptive system results to the EQ-5D utility index [42].

SF-36 assesses HRQoL in eight dimensions related to the physical and mental components of health. It is possible to synthesize the information obtained with the eight domains into two summary scores, one specific for physical health (Physical Summary Score - PCS)and the other for mental health (Mental Summary Score - MCS): the higher the score, the better the component of HRQoL measured [37,43]. For this study, PCS and MCS results will be shown.

Data on both antipsychotic and concomitant *drug treatments* were taken, recording information on type of drug, dosing, duration of treatment and details on switches or interruptions.

*Compliance and attitude toward antipsychotic treatment* at the time of each examination were assessed with the 30-item version of Drug Attitude Inventory (DAI-30) [44], a scale self-completed by the patients to measure their subjective response to medication as well as their attitude towards pharmacological treatment, illness and health. We computed the score on 25 items which, according to recent research, mostly contribute to increase the score’s internal consistency [45]: accordingly, the score ranges from a minimum of 25 (negative attitude) to a maximum of 50 (the higher the score, the more positive the attitude). Patients’ compliance was also assessed according to the physicians’ opinion: these were asked to specify whether the patient took the antipsychotic treatment according to prescription, with 6 possible answers: always (81-100% of times), frequently (61-80% of times), sometimes (41-60% of times), very rarely (21-40% of times), never (≤20% of times), or not applicable if it was the first time for the physician to see that patient.

*Persistence* with antipsychotic treatment was assessed by calculating the number of days the patients were in either atypical or typical class treatment until interruption or switch to an alternative class of drugs. We also calculated the proportion of patients who switched at least once, during the follow up period, and the number of switches occurring among the atypical, typical, combined regimen (any typical or atypical), or no treatment.

Finally, we calculated the occurrence of relapse, identified in terms of admittance to hospital or residential care units during the follow up period.

### Direct and indirect costs

*Direct costs* were assessed with the information obtained from the physician on the following medical resource consumption: pharmacological treatment with antipsychotics, concomitant drug treatment, psychotherapy, hospital admissions in both full-day and/or day-hospital, admissions in residential care units, nurse home visits, specialist medical examinations, laboratory and instrumental tests. Direct costs were quantified in monetary terms by multiplying the amount of the resource consumed by the corresponding unit cost, according to prices and tariffs applied in Italy in 2007. Both the patients’ and the National Health Service (NHS) perspectives were adopted to estimate these costs, according to the actual payer. For costs paid by the NHS, pharmacological treatment unit costs were obtained from the Italian Drug Agency price list [46]. Costs of hospital admissions were calculated according to the Diagnosis Related Group (DRG) regional tariffs [47]. Costs for nurse home visits, diagnostic tests, medical specialist examinations and psychotherapy were obtained from the regional outpatient lists [48]. Finally, a specific tariff was used to assign costs for access to residential care units, structured psychosocial rehabilitation groups or day centres [49]. If patient out-of-pocket costs were incurred, the information reported by patients was used. However, the NHS in Italy is responsible for providing and paying for most of the health care costs to manage individuals with chronic conditions (schizophrenia), hence we did not expect to estimate high costs paid by the patients for these costs.

*Indirect costs* were estimated by calculating patients’ and their caregivers’ loss of productivity for reasons attributable to the target condition. The estimate of loss of productivity is not easy to perform in this population: some patients were still students, while others did not complete even their compulsory education, probably for reasons related to their condition, which must be considered as a loss of productivity and possible cause of future loss. Other patients were performing unpaid work, like an intern or apprenticeship, obtained because of and according to their condition. In order to avoid a biased monetary estimate of productivity loss based on national working tariffs and standard methods (e.g. human capital approach [50]), which do not take into account the cost attributable to the loss of the opportunity to study and/or work due to the illness, we preferred not to assign a monetary value but rather to assess this component of cost of illness according to the following method. First – the frequency of idle patients was estimated: according to their age ranging from 18 to 40 years, all the patients were expected to be students, involved in paid or unpaid job, or at least housewives. If they were not doing any of these activities, they were classified as idle for reasons attributable to their clinical condition. Second - regardless of their working status, patients were also asked if they lost days of work, school or in doing any usual activity (e.g. housekeeping) for reasons attributable to their condition. Loss of productivity was estimated also for the patients’ caregivers: information on their job and on the number of days they lost from work/study/usual activities was collected.

### Statistical analyses

The sample socio-demographic, clinical and HRQoL characteristics were described using proportions for categorical data, mean and/or median as central tendency parameters for continuous data, standard deviation (SD), minimum (min) and maximum (max) values as dispersion parameters. The persistence with the antipsychotic drug treatment was estimated by means of Kaplan-Meier curves, by comparing the mean number of patient-days (i.e. number of days each patient persisted in each treatment) of permanence in the same drug class (i.e. class of atypical, typical, combination of both classes, or no antipsychotic drug).

Relapse was calculated as the proportion of patients with at least one relapse event during follow up and as the mean and SD number of patient-days free from relapse.

Consumption of health care resources is expressed as the proportion of patients consuming each health care item, and as mean (min-max) number of examinations, days, sessions per patient-month. Monetary values are reported as mean €/patient-month. Indirect costs are reported in terms of proportions of idle patients, of patients and caregivers losing at least 1 day of productivity, and as mean (min-max) days/patient-month or days/caregiver-month of productivity lost. The use of mean days per patient-month makes it possible to adjust the results for the different periods of time between the examinations when the data were collected. Because of the highly skewed distribution of cost variables, we report the distribution of both costs and days of productivity lost per patient per month as a variability measure.

Data collected at each examination (at enrolment, and 12 ± 2, 36 ± 2 and 52 ± 2 weeks later) were analysed and reported to show the trend found during the observational period. To compute the means of the variables, the number of patients (when assessing e.g. clinical status, HRQoL, compliance, rate of idle patients) or the number of patient-months (in the assessment of costs occurring during the reference period) available at each examination were used as denominators.

Comparisons between naïve and non naïve patients were conducted. To compare baseline characteristics, between-group testing was performed with independent sample t-tests for continuous variables and Chi-square tests or Fischer exact tests for categorical variables. Two-way repeated measures ANOVA (with time set as repeated factor) was used to assess the trend of the measurements during the observational period on the full sample and to compare the trends between naïve and non-naïve patients. A Greenhouse-Geisser correction was applied if the assumption of sphericity was not respected. If the trend was statistically significant (p < 0.05), multiple comparisons using the Bonferroni correction were performed to assess the differences between each pair of examinations. All analyses were performed using SPSS version 18.0 software (SPSS, Chicago, IL).

## Results

### Sample description

A total of 661 patients were enrolled in 86 Mental Health centres scattered all over Italy, during 2006 and 2007: 24 patients were identified to be not eligible for participation in the study, so the valid study sample included 637 patients at baseline.

Among the valid patients, at enrolment, 90 (14.1%) were visiting the centre for the first time, while 124 (19.5) were in need of a treatment change or initiation (12 out of the 124 patients had not taken any treatment in the previous 90 days). Overall, 63 patients (9.9%) were identified as naïve, as they were both visiting the centre for the first time and starting a new treatment regimen.

Tables 1 and 2 show the description of the full sample characteristics at baseline, and also report differences between naïve and non naïve patients.

**Table 1:** Patients’ socio-demographic characteristics at enrolment (N = 637)

**Description**	**Values**	**Non naïve (N = 574)**	**Naïve (N = 63)**	**p-value**
**Gender:**				
Males, n (%)	414 (65.0)	374 (65.2)	40 (63.5)	0.793*
**Age** (years)**:**				
Median (min-max)	31.0 (18–40)	32.0 (18–40)	28.0 (18–40)	<0.0001^‡^
Mean (SD)	30.9 (5.49)	31.2 (5.4)	28.3 (5.9)	
**Body Mass Index:**				
Mean (SD)	27.2 (5.3)	27.4 (5.4)	25.2 (4.3)	0.002^‡^
**Education:**				
Primary school, n (%)	27 (4.2)	25 (4.4)	2 (3.2)	0.164**
Lower secondary school, n (%)	301 (47.3)	277 (48.3)	24 (38.1)	
Upper secondary school, n (%)	271 (42.5)	240 (41.8)	31 (49.2)	
Graduate/post-graduate, n (%)	38 (6.0)	32 (5.6)	6 (9.5)	
**Marital status:**				
Single, n (%)	546 (85.7)	494 (86.1)	52 (82.5)	0.576**
Married, n (%)	66 (10.3)	57 (9.9)	9 (14.3)	
Divorced/separated, n (%)	24 (3.8)	22 (3.8)	2 (3.2)	
Widow, n (%)	1 (0.2)	1 (0.2)	0	
**Family and caregiver:**				
Patients living alone, n (%)	28 (4.4)	25 (4.4)	3 (4.8)	0.751**
Patients with a caregiver, n (%)	309 (48.5)	299 (52.1)	29 (46.0)	0.619*
**Working status:**				
Idle, n (%)	336 (52.8)	313 (54.5)	24 (38.1)	0.100**
Working^$^, n (%)	225 (35.3)	200 (34.8)	24 (38.1)	
Student, n (%)	60 (9.4)	48 (8.4)	12 (19.0)	
Housewife, n (%)	16 (2.5)	13 (2.3)	3 (4.8)	
**Economic support for schizophrenic condition:**				
Disability pension, n (%)	187 (29.4)	185 (32.2)	2 (3.2)	<0.0001**
Sickness benefit, n (%)	7 (1.1)	7 (1.2)	0	
Both, n (%)	5 (0.8)	5 (0.9)	0	

* A Chi-square test was applied.

** A Fisher’s exact test was applied.

‡ An independent sample Student’s *t*-test was applied.

$The term “working”refers to subjects with paid work and unpaid work (e.g., intern, apprenticeship).

**Table 2:** Patients’ clinical characteristics and HRQoL at enrolment (N = 637)

**Description**	**Values**	**Non naïve (N = 574)**	**Naïve (N = 63)**	**p-value**
**Diagnosis:**				
Schizophrenia, n (%)	549 (86.2)	510 (88.9)	39 (61.9)	<0.0001*
Schizophreniform disorder, n (%)	88 (13.8)	64 (11.1)	24 (38.1)	
**Type of schizophrenia**:				
Catatonic, n (%)	7 (1.3)	7 (1.4)	0	0.661**
Disorganized, n (%)	56 (8.8)	51 (10.0)	5 (12.8)	
Paranoid, n (%)	372 (67.8)	345 (67.6)	27 (69.2)	
Undifferentiated, n (%)	88 (16.0)	81 (15.9)	7 (17.9)	
Residual, n (%)	26 (4.1)	26 (5.1)	0	
**Duration of illness**(years):				
Mean (SD)	3.7 (3.0)	4.0 (3.0)	1.2 (2.5)	<0.0001^‡^
**Diagnosis:**				
<1 year before, n (%)	175 (27.5)	128 (22.3)	48 (76.2)	<0.0001*
1–5.99 years before, n (%)	284 (44.6)	273 (47.6)	9 (14.3)	
6–10 years before, n (%)	178 (27.9)	173 (30.1)	6 (9.5)	
**Age at onset of psychotic symptoms**(years)**:**				
Mean (SD)	24.2 (5.5)	24.1 (5.5)	24.6 (5.9)	0.576^‡^
**Age at first antipsychotic treatment**(years)**:**				
Mean (SD)	25.5 (5.4)	25.5 (5.3)	26.1 (6.0)	0.370^‡^
**Age at first hospitalization**(years)**:**				
Mean (SD)	26.2 (5.4)	26.2 (5.4)	25.5 (5.8)	0.589^‡^
**PANSS score**				
**Positive subscale**, Mean (SD)	17.3 (7.4)	16.9 (7.2)	21.6 (7.9)	<0.0001^‡^
**Negative subscale**, Mean (SD)	23.7 (8.5)	23.5 (8.5)	25.7 (8.3)	0.050^‡^
**General subscale**, Mean (SD)	45.6 (14.6)	44.8 (14.6)	52.7 (13.5)	<0.0001^‡^
**Total**, Mean (SD)	86.6 (27.4)	85.2 (27.1)	100.0 (26.2)	<0.0001^‡^
**GAF score:**				
Mean (SD)	54.1 (13.8)	54.6 (13.9)	49.6 (12.6)	0.007^‡^
**CGI-S score:**				
Mean (SD)	4.3 (2.2)	4.3 (1.1)	4.6 (0.9)	0.011^‡^
**EQ-5D VAS**				
Mean (SD)	63.5 (17.9)	63.5 (17.9)	63.5 (18.2)	0.991^‡^
**EQ-5D utility score**				
Mean (SD)	0.71 (0.3)	0.70 (0.3)	0.72 (0.3)	0.656^‡^
**SF-36 PCS**				
Mean (SD)	47.5 (9.3)	47.5 (9.4)	47.4 (9.2)	0.928^‡^
**SF-36 MCS**				
Mean (SD)	39.0 (9.6)	39.3 (9.5)	36.6 (10.4)	0.049^‡^

* A Chi-square test was applied.

** A Fisher’s exact test was applied.

‡ An independent sample Student’s *t*-test was applied.

Fifty patients, 7.8% of the study sample, were not observed until the end of the study for various reasons: 614 came back for the first follow-up examination, 603 underwent the second follow-up examination, and 587 patients were observed for the full observational period. As regards the reason for discontinuation, 39 patients (6.1%) asked their clinician to withdraw from the study, while 5 had reasons not allowing them to remain in the study (pregnancy, death, severe adverse event), 5 (0.8%) moved to a different city (3) or different care centre (2), and 1 patient was lost to follow-up. There were no relevant differences between the patients who completed the follow-up and those who discontinued in socio-demographic aspects, clinical characteristics and HRQoL. According to these data, we do not have elements to suspect that discontinuations were related to patients’ outcomes and that could have biased our results.

Overall, the patients were observed for a mean of 14.4 (3.0-17.9) months, considering both the retrospective and prospective period.

During follow up, 109 patients, 17.1% of the study sample had symptom relapses. On average, 460.2 (SD = 5.7) days/patient free from symptom relapse were estimated through the full observational period.

### Drug treatment

**Figure 1:**
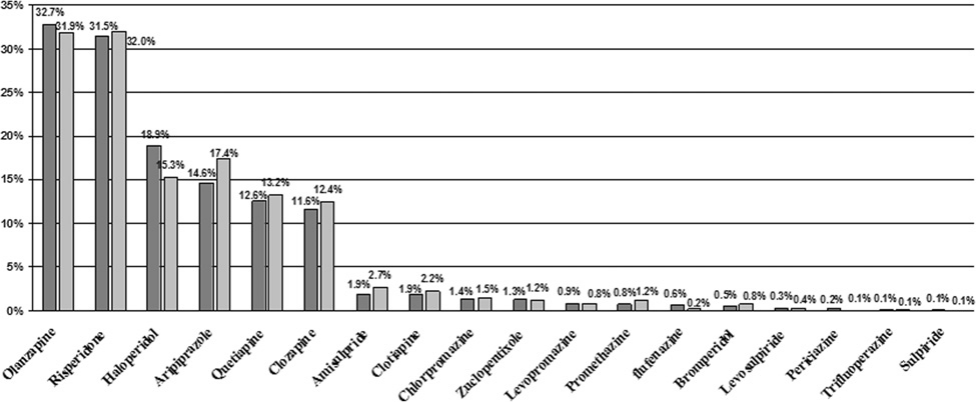
**Antipsychotic drug treatment during the 90 days before enrolment and during prospective follow up period.** Percentages refer to the patients taking the drug at least once during the 90 days before enrolment (dark grey bars) and/or during the prospective follow up period (light grey bars), either alone or with other antipsychotic drugs. Percentages reported do not necessarily sum up to 100%.

Patients were treated with several different antipsychotics (18 drugs in total) and concomitant therapies. Concomitant therapy mostly consisted of anxiolytics, used by approximately 40% of the patients, antidepressants, used by 30%, anticholinergics, used by 11%, and antiepileptic medications, used by 14% of the patients over the observational period. As regards antipsychotic drugs, during both the 90 days before enrolment and the prospective follow up period, one third of the study sample took olanzapine and one third risperidone; the other most frequently used drugs were haloperidol, aripiprazole, quetiapine and clozapine (Figure 1).

At enrolment, the patients reported a mean DAI score of 43.4, with a minimum of 25 (1 patient, 0.2%) and a maximum of 50 (45 patients, 7.1%). With the exclusion of 90 patients, for whom this information was not available at enrolment as they were attending the centre for the first time, the physicians reported that 71.1% (out of 547 patients) always took the prescribed antipsychotic therapy, 23.2% took it very frequently, while 4.0% took the therapy sometimes, 1.3% very rarely and 0.4% never took the prescribed therapy.

During the 90 days before enrolment, 58.6% of the 637 patients took only atypical antipsychotics, 2.8% only typical antipsychotics, 6.0% a combination of both atypical and typical, and 1.7% never started an antipsychotic treatment. In that period, 30.9% of the study sample switched at least once to an atypical, typical, combination or no antipsychotic treatment.

**Figure 2:**
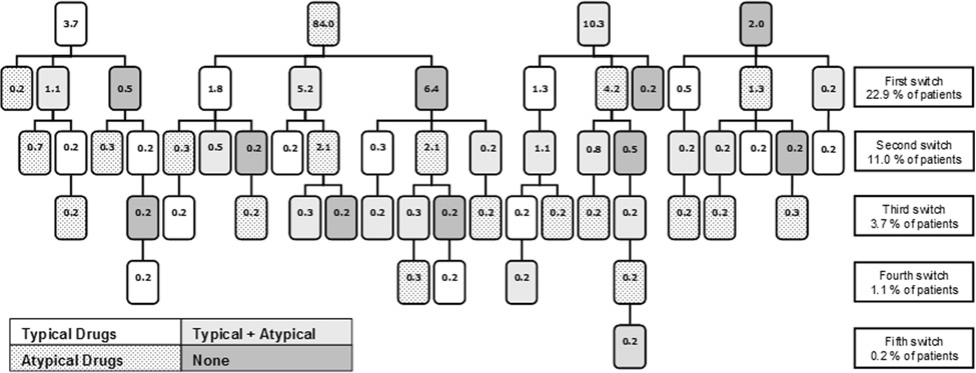
**Antipsychotic drug treatment and switches taken from enrolment during the follow up period.** Each box reports the percentage of patients taking one out of the four treatment options (typical, atypical, combination of typical and atypical, none). Vertical lines joining the boxes represent the switch from one treatment option to the alternative option(s). Horizontal lines join the number and the type of options to which treatment change occurred in each step of switch. Boxes on the right of the figure report the percentage of patients involved in each step of treatment switch.

Antipsychotic drug treatment used after the enrolment examination included atypical drugs, started in 84% of the patients. During follow-up 13.4% of these patients switched to typical, combined or no treatment (Figure 2). Overall, 22.9% of the study sample switched their treatment (class of drugs) at least once, 11% switched at least twice, while 1.3% switched 4 or 5 times.

**Figure 3:**
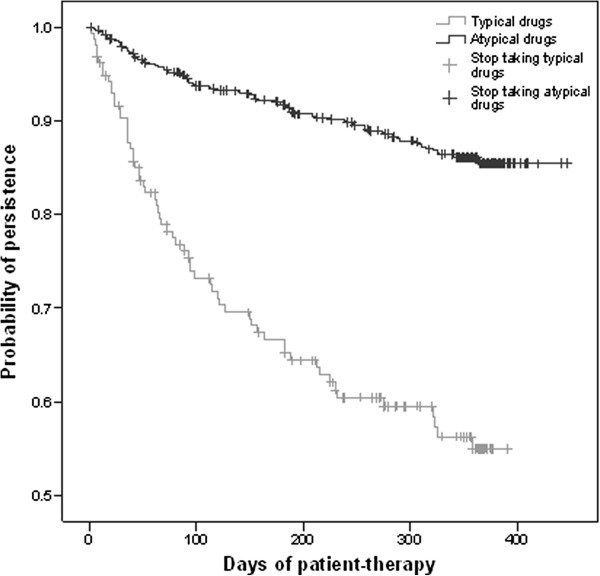
**Persistence with antipsychotic drug treatment from enrolment to endpoint or to patients’ withdrawal from the study.** The graph shows the probability of persistence (Y axis) during the therapy period (X axis) with the treatment drug class (black curve for atypical and grey curve for typical).

During follow-up, the persistence with atypical antipsychotics was higher than the persistence with typical antipsychotic therapy (Figure 3): on average, 402.8 patient-days were estimated for atypical antipsychotic treatment, and 263.0 patient-days for typical treatment.

### Other health care resource consumption

Table 3 shows details on the proportion of patients and amount of other health care resources consumed during the observational period. Overall, 24% of the patients received psychotherapy before enrolment, for an overall mean of 1.7 days/patient-month, with a decreasing trend recorded during the follow-up period.

**Table 3:** Consumption of health care services

**Type of resource**	**From −90 days to enrolment (N = 637)**	**From enrolment to follow-up examination1 (N = 614)**	**From follow-up examination 1 to 2 (N = 603)**	**From follow-up examination 2 to 3 (N = 587)**
**Psychotherapy**				
*Any type*				
- Proportion of patients	24.3%	22.8%	20.9%	17.4%
- No. sessions/pat-month, Mean (min-max)	1.7 (0.0-48.0)	1.6 (0.0-51.4)	1.6 (0.0-69.5)	1.3 (0–129.3)
*Individual, group, familiar psychotherapy*				
- Proportion of patients	15.9%	14.0%	12.6%	12.1%
- No. sessions/pat-month, Mean (min- max)	0.7 (0.0-48.0)	0.5 (0.0-51.4)	0.6 (0.0-68.6)	0.5 (0.0-62.6)
*Structured psychosocial rehabilitation group*				
- Proportion of patients	6.8%	7.0%	6.6%	2.7%
- No. sessions/pat-month, Mean (min-max)	0.6 (0.0-25.7)	0.5 (0.0-20.3)	0.5 (0.0-27.5)	0.4 (0.0-66.7)
*Day center*				
- Proportion of patients	4.9%	5.9%	5.5%	4.8%
- No. sessions/pat-month, Mean (min-max)	0.5 (0.0-24.0)	0.5 (0.0-22.9)	0.5 (0.0-23.5)	0.4 (0.0-24.1)
**Residential care units**				
Proportion of patients	1.7%	2.0%	2.5%	2.7%
- No. any accesses	15	13	15	20
- No. accesses for symptom relapse	6	6	8	6
- No. days/pat-month, Mean (min-max)	0.25 (0–28.0)	0.32 (0–28.2)	0.53 (0–29.7)	0.6 (0.0-29.8)
**Hospital admissions (full day or day hospital)**				
- Proportion of patients for any reason	11.9%	7.2%	6.6%	8.4%
- No. admissions/pat-month, Mean (min-max)	0.05 (0.0-1.7)	0.03 (0.0-1.0)	0.03 (0.0-2.5)	0.02 (0.0-1.3)
- Proportion of patients for symptom relapse	9.7%	4.7%	4.2%	6.5%
- No. admissions/pat-month, Mean (min-max)	0.03 (0.0-1.0)	0.02 (0.0-1.0)	0.02 (0.0-0.9)	0.01 (0.0-0.7)
**Nurse home visits**				
- Proportion of patients	7.4%	7.2%	5.1%	4.6%
- No. visits/pat-month, Mean (min-max)	0.6 (0.0-60.0)	0.2 (0.0-64.3)	0.1 (0.0-7.8)	0.1 (0.0-8.3)
Specialist examinations*				
- Proportion of patients	9.8%	11.7%	12.3%	16.2%
- No. examinations/pat-month, Mean (min-max)	0.1 (0.0-23.3)	0.1 (0.0-4.5)	0.9 (0.0-3.6)	0.1 (0.0-11.9)
Diagnostic tests**				
- Proportion of patients	29.0%	25.2%	24.2%	35.8%
- No. tests/pat-month, Mean (min-max)	1.4 (0.0-40.0)	1.0 (0.0-23.0)	1.0 (0.0-18.0)	1.8 (0.0-27.7)

* More often psychiatrist, cardiologist, neurologist, gynaecologist or dietician. ** Blood tests and/or instrumental tests.

One criterion of eligibility was not staying at residential care units at the time of enrolment: however, 1.7% of the patients accessed this service during the previous 90 days, for an average of 0.25 days/patient-month. Furthermore, during the follow up period after enrolment, the use of this resource increased in terms of both number of accesses (up to 20) and number of patients involved (2.7%), hence in terms of total duration of staying. In contrast, hospitalizations, mostly attributable to symptom relapse, decreased on average, together with nurse home visits. The specialist examinations did not show a well defined trend during the follow-up.

### Direct costs

As expected, the quota paid by most of the patients was null, hence the amounts reported can be considered as corresponding to the amount paid by the NHS. Total direct costs corresponded to an average of 408 €/patient-month (Table 4) and were stable overall during the observational period (F(2.3,1755) = 0.2, P = 0.842). In particular, 81.2% of the patients cost less than 500 €/patient-month, 9.9% cost between 500 and less than 2,000 €/patient-month, while only 3.0% cost from 2,000 to 9,500 €/patient-month. The cost driver was the pharmacological treatment, corresponding to 30-36% of total medical costs. However, some cost items varied differently during the observational period: cost of treatment with antipsychotic drugs and for accessing residential care units increased, while psychotherapy and hospital admissions decreased (Table 4).

**Table 4:** Direct medical costs

**Variable description**	**90 days before enrolment (N = 637)**		**From enrolment to follow-up examination 1 (N = 614)**		**Between follow-up examinations 1 & 2 (N = 603)**		**Between follow-up examinations 2 & 3 (N = 587)**	
	**Mean €/pat-month**	**%**	**Mean €/pat-month**	**%**	**Mean €/pat-month**	**%**	**Mean €/pat-month**	**%**
Drug treatment	115.30	29.5	144.9	35.2	146.5	34.2	144.57	35.9
*Antipsychotic drugs*	*100.70*	*25.8*	*127.9*	*31.1*	*129.0*	*30.2*	*127.83*	*31.7*
*Concomitant drugs*	*14.57*	*3.7*	*16.8*	*4.1*	*17.0*	*4.0*	*16.73*	*4.1*
Psychotherapy	114.00	29.2	108.36	26.3	98.20	22.9	93.53	23.2
Hospitalizations, of which	105.75	27.1	93.33	22.7	91.69	21.4	69.62	17.3
*Full day admissions for symptom relapse*	*85.51*	*21.9*	*42.76*	*10.4*	*38.87*	*9.1*	*34.14*	*8.5*
Residential Care Units	37.22	9.5	49.11	11.9	80.60	18.8	85.06	21.1
Nurse home visits	9.21	2.4	10.02	2.4	5.46	1.3	4.67	1.2
Lab and instrumental diagnostic tests	6.00	1.5	3.87	0.9	3.49	0.8	3.40	0.8
Specialist examinations	2.91	0.7	1.97	0.5	2.35	0.5	2.32	0.6
**Total medical costs**	**390.39**	**100**	**411.61**	**100**	**428.27**	**100**	**403.17**	**100**

The trend of direct costs found can be ascribed to two main reasons: first – the decision to exclude patients living in residential care units at enrolment caused lower costs imputable to this reason, until the enrolment examination. Later, during the follow-up, the patients accessed these units and stayed for up to 20 days/patient-month. The daily cost of staying is high (on average 151 € per day), which, multiplied by the long stays, contributed to a considerable portion of the total costs. Second – 9.9% of the patients were naïve at enrolment: until then the cost for antipsychotic drug treatment of these patients was 28 €/patient-month (10 out of these 63 patients did not receive any treatment during the pre-enrolment period), as compared with 108 €/patient-month spent for non naïve patients, but reached 112.19 €/patient-month after one year. On the other hand, the cost for hospital admissions of naïve patients was 104 €/patient-month, reduced to 39.76 €/patient-month one year later; and that for psychotherapy was 45.70 €/patient-month, decreased to 8.21 €/patient-month one year later. However, total direct costs were not significantly different between naïve and non naïve patients, during the observational period (F(2.3,1755) = 0.1, P = 0.964).

### Indirect costs

Throughout the entire observational period, 62.2% of the patients did not generate any loss in days of productivity, for themselves or for their caregivers. Up to 5 days/patient-month were lost by 28.7% of the patients, and 5–10 days/patient-month by 6.0%, while 10–31 days/patient-month lost involved 3.2% of the patients.

The percentage of idle patients and of patients and caregivers losing days of productivity decreased during the follow up period. The mean number of days of productivity lost also decreased among patients and caregivers losing at least 1 day of productivity. As a result, among the entire study sample, while an average of 3.5 days/patient-month was lost during the 90 days before enrolment by patients and caregivers, this amount decreased to less than 1 day/patient-month 1 year later (Table 5).

**Table 5:** Loss of productivity among patients and their caregivers*

**Variable description**	**90 days before enrolment (N = 637)**	**From enrolment to follow-up examination 1 (N = 614)**	**From follow-up examination 1 to 2 (N = 603)**	**From follow-up examination 2 to 3 (N = 587)**
Frequency of idle patients	52.8%	51.3%	50.8%	49.3%
Frequency of patients losing ≥1 day of productivity	23.3%	8.5%	7.3%	9.4%
Frequency of caregivers losing ≥1 day of productivity	18.6%	8.7%	11.0%	11.0%
No. days/patient-month of productivity loss *(computed among the whole study sample)*	2.9 (0.0-30.0)	1.0 (0.0-30.0)	0.6 (0.0-30.0)	0.6 (0.0-30.0)
No. days/patient-month of productivity loss *(computed among patients loosing productivity)*	12.6 (0.3-30.0)	12.0 (0.3-30.0)	8.4 (0.3-30.0)	6.2 (0.4-30.0)
No. days/caregiver-month of productivity loss *(computed among all caregivers in the study sample)*	1.2 (0.0-30.0)	0.2 (0.0-7.5)	0.5 (0.0-28.1)	0.2 (0–5.4)
No. days/caregiver-month of productivity loss *(computed among caregivers loosing productivity)*	6.3 (1.0-30.0)	2.8 (0.3-7.5)	4.1 (0.6-28.1)	1.5 (0.4-30.0)
No. days/patient-month of total productivity loss by both patients and caregivers *(computed among patients study sample)*	3.5 (0.0-60.0)	1.1 (0.0-31.7)	0.8 (0.0-33.7)	0.7 (0.0-30.0)

* values for days/person-month are mean values (min-max).

Interestingly, among naïve patients, 7.4 days/patient-month of productivity were lost by both the patients and their caregivers before enrolment, while a reduction was detected during follow-up, reaching 0.7 days/patient-month. The other patients and their caregivers lost 3.1 days/patient-month before enrolment, with a reduction to 0.7 days/patient-month during follow-up. The decreasing trend of productivity lost by all patients and caregivers was statistically significant (F(1.8,1755) = 41.3, P < 0.0001) and was significantly different between naïve and non naïve patients (F(1.8,1755) = 7.8, P = 0.001). In particular, in both the subgroups there was a significant decrease in productivity lost between the enrolment examination and the 3 follow up examinations (p < 0.001)

### Outcomes during the follow-up

During the follow-up, the mean DAI-30 score remained more or less stable in both naïve and non naïve patients, although the naïve patients showed a slightly lower mean level of attitude toward treatment (Table 6). Furthermore, according to the clinicians, most of the patients did not change their attitude or compliance toward treatment: in particular, compared with the baseline, after one year (follow-up examination 3) 70.5% did not change their compliance, 14.4% improved it, while 15.2% worsened their compliance with respect to the year before.

**Table 6:** Trends on clinical outcomes, HRQoL and attitude toward treatment

	**DAI total score Mean (SD)**		**PANSS total score Mean (SD)**		**GAF Mean (SD)**		**CGI-S Mean (SD)**		**EQ-VAS Mean (SD)**		**EQ-utility Mean (SD)**		**SF-36 PCS Mean (SD)**		**SF-36 MCS Mean (SD)**	
	**Non naïve**	**Naïve**	**Non naïve**	**Naïve**	**Non naïve**	**Naïve**	**Non naïve**	**Naïve**	**Non naïve**	**Naïve**	**Non naïve**	**Naïve**	**Non naïve**	**Naïve**	**Non naïve**	**Naïve**
Enrolment visit	43.9 (5.0)	41.0 (5.8)	85.5 (27.2)	98.2 (26.1)	54.6 (13.9)	49.9 (13.0)	4.2 (1.1)	4.6 (0.9)	63.7 (18.0)	63.3 (18.4)	0.71 (0.3)	0.68 (0.3)	47.8 (9.2)	48.3 (9.6)	39.5 (9.6)	37.1 (10.9)
Follow-up Examinatio2	43.9	41.7	79.8	81.1	57.1	58.6	4.1	3.9	66.2	67.8	0.74	0.76	48.2	48.7	40.6	39.8
	(5.0)	(4.8)	(27.4)	(25.7)	(13.7)	(12.6)	(1.1)	(1.2)	(16.8)	(14.6)	(0.3)	(0.2)	(9.5)	(9.0)	(9.3)	(9.5)
Follow-up examination3	44.2	42.1	75.3	76.1	59.4	62.4	3.9	3.7	68.0	72.2	0.75	0.80	49.1	51.9	41.5	42.0
	(4.8)	(5.1)	(27.0)	(24.5)	(14.3)	(12.0)	(1.2)	(1.1)	(16.6)	(14.4)	(0.3)	(0.2)	(9.3)	(8.1)	(8.9)	(8.0)
Follow-up examination4	44.2	42.6	71.4	66.9	61.4	67.6	3.8	3.4	70.5	75.5	0.79	0.81	49.7	53.5	41.9	43.0
	(4.8)	(5.2)	(25.8)	(21.1)	(14.2)	(11.5)	(1.2)	(1.1)	(16.6)	(14.7)	(0.2)	(0.2)	(8.9)	(6.6)	(8.9)	(10.0)
Time effect*	F(2.7,1254) = 2.7 P = 0.053		F(2.3,1752) = 156.9 P < 0.0001		F(2.4,1752) = 132.6 P < 0.0001		F(2.5,1752) = 68.9 P < 0.0001		F(2.7,1581) = 25.9 P < 0.0001		F(2.8,1569) = 10.9 P < 0.0001		F(2.8,1434) = 14.1 P < 0.0001		F(2.8,1434) = 17.1 P < 0.0001	
Time effect between subgroups*	F(2.7,1254) = 1.1 P = 0.360		F(2.3,1752) = 22.7 P < 0.0001		F(2.4,1752) = 25.9 P < 0.0001		F(2.5,1752) = 14.3 P < 0.0001		F(2.7,1581) = 2.3 P = 0.080		F(2.7,1569) = 1.6 P = 0.192		F(2.8,1434) = 3.5 P = 0.017		F(2.8,1434) = 3.0 P = 0.033	

*Time effect refers to the differences estimated between mean observations at any pair of examinations, during the observational period, on the full sample.

§ Time effect estimated comparing two subgroups: non naïve versus naïve patients.

On the other hand, the patients’ clinical status measured by the physicians with the PANSS, the GAF and the CGI-S scales, and patients’ HRQoL as reported by the patients themselves, showed on average a significant (P < 0.0001) improvement during follow-up (Table 6), suggesting that the treatment strategy adopted in these patients was beneficial to their health, according to both physician’s and patient’s points of view.

In particular, the naïve patients had an average higher improvement than the non naïve patients, which was statistically significant in the SF-36 PCS and MCS scores (Table 6). Among the non naïve patients, significant (p < 0.05) improvements were found between each pair of examinations, in the CGI-S, GAF, PANSS and EQ-5D VAS mean scores. However, the PCS mean score change was not significant between enrolment and follow-up examination 1, and between follow-up examination 2 and examination 3, and the MCS mean score change was not significant between follow-up examinations 2 and 3. Finally, we found a significant improvement with the EQ-5D utility score in all pairs of examinations except between enrolment and follow-up examination 1, and between follow up examination 1 and 2.

In the naïve group, there were significant improvements (p < 0.05) in GAF and PANSS total mean score comparisons between at least two examinations. While significant improvements were observed between follow-up examinations 1 and 2 in the CGI-S score, between enrolment and follow-up examination 3 in the EQ-5D VAS, PCS and MCS scores, between enrolment and follow-up examination 2 in the EQ-5D VAS and MCS scores, and between follow-up examination 1 and examination 3 in the EQ-5D VAS and PCS scores. No significant improvements were found in the EQ-5D utility score.

## Discussion

The novelty of the present study is its ability to provide the currently available most complete picture on the socio-economic burden and outcomes on young patients with recent diagnosis of schizophrenia or schizophreniform disorder, observed in a context of real clinical practice.

The study showed how these patients received several and complex treatments to manage their condition, using the many options available in the healthcare system. In particular, many antipsychotic drugs and concomitant therapies were used in different combinations. As regards the antipsychotic drug treatment, we found a higher persistence for the treatment with atypical antipsychotic drugs (more than 400 patient-days) than for typical drugs (263 patient-days). In this study we saw also how clinical and perceived health can change (improve) in nearly one year, according to the treatment strategies adopted. The full sample showed an overall improved trend in both clinical and perceived health (i.e., HRQoL): however, this improvement was on average higher among the naïve patients, i.e., those patients that were seen at the participating centres and started to receive health care starting from their enrolment in the study. Not only did clinical outcomes and HRQoL improve on average, but also the patients’ productivity. Furthermore, while no significantly different overall direct costs trends were found between naïve (i.e., patients that had started or changed antipsychotic treatment recently) and non naïve patients, naïve patients showed generally a significant mean higher improvement of clinical outcomes, HRQoL and indirect costs, compared to the others. However, the study sample was probably not suitable to obtain reliable results from these comparisons. Our results have a trend similar to that found in other studies, where treatment is associated with improvement in HRQoL [17,18] and clinical outcomes. [19-21]. A similar trend was found also by Strakowsi et al.[24], who showed that among patients with newly onset schizophrenic disorder, treatment is associated with improvements in most HRQoL domains, measured with the SF-36 instrument. Other studies show that outcomes in patients with a first-episode psychosis may be improved by an early intervention treatment and reduction of the untreated period [22,23,25-27] but long term effects are still not clear [26-28].

In our study sample, a quarter of medical costs are attributable to antipsychotic drug treatment, followed by costs for psychotherapy and for hospital admissions: these results are different from those obtained in previous research, where it has been shown that the highest proportion of direct costs is attributable to in-patient care, while drug treatment generates lower costs [7-10,16]. However, it must be noted that the data available in the literature refer to different populations from different countries, where different treatment modalities could be applied, or different unit costs are applicable, or which include older patients than those involved in our study [6-11,13-15,51,52]. In our study, medical costs during the follow up period remained stable overall. However, while some cost items increased (antipsychotic drug treatment, admission to health care units), others decreased (mainly hospital costs for symptom relapses), which could have depended also on the patients’ inclusion criteria at enrolment. Previous studies have demonstrated that loss of productivity is the main component of overall cost of schizophrenia [6-10,13-15,51,52]. Our results confirm the high productivity loss among patients and their family caregivers, measured in terms of frequency of idle patients and in terms of days lost from productivity. However, we also observed a reduction of productivity loss during the follow up. This point is of great interest from the societal point of view, since schizophrenia is associated with poverty and homelessness, thus representing a significant amount of resource costs to deal with these problems. The National Institute for Health and Clinical Excellence (NICE) has recently recommended taking into account wider societal costs, including productivity losses of people with schizophrenia and of their family caregivers’, when cost-effectiveness of treatment is assessed [52]. It must be noted that it is not easy to also include loss of productivity in the computation of costs [9], and this loss often remains excluded from calculations: for instance, Serretti and co-workers highlighted its importance, but did not include this parameter in their Italian simulation of the socio-economic burden of schizophrenia [53]. Palazzolo et al. [54] pointed out that the combination of early diagnosis and the use of atypical medications would change the face of schizophrenia, allowing many patients to start working again. Our findings suggest that appropriate therapy reduces loss of productivity in a relatively short time period and that it may also promote an increment of the working/job inclusion of schizophrenia patients, improving in turn their social networks.

The treatment pattern observed in this study, which required several switches between drugs, demonstrates how treating patients with schizophrenia requires the availability of a wide and complex armamentarium of products (e.g., drugs) and services (e.g., psychosocial treatment, psychotherapy, etc.), likely involving different interacting factors, such as clinical severity, patient compliance, service accessibility [55]. In this regard, the most recent NICE guidelines [52] report that choosing the most appropriate drug and formulation according to each patient’s needs and characteristics might be more important than only taking into account the main recognized properties of the different classes of drugs. In this way, NICE acknowledges the importance of a personalized treatment and highlights the importance of high levels of adherence to antipsychotic treatment to reduce the risk of relapse and further hospitalization costs.

Our study has some potential limitations: first, there was no control group in the present study, hence, we could not verify if the trends estimated with the different measures completed by the patients or the clinicians might actually represent a practice effect, i.e., an increase in the test scores from one administration to the next without any intervention. However, in a recent study aimed at investigating practice effects on a battery of scales, this was not found on the PANSS and on a visual analogue scale analysing the HRQoL [56]. Second, although we could preliminarily estimate the possible incremental benefit attributable to the treatment applied on naïve patients, our sample size was too small to draw reliable results and conclusions. However, with regard to these two limits, this study was not designed for making comparisons between different patients, but rather to observe treatment strategies adopted in clinical practice and related consequences on patients’ health and costs to society. Third, the study may suffer from a selection bias, as patients who participated may be more likely to comply with medication than the overall target population. Fourth, our results on the higher persistence for atypical versus typical drugs classes could partially depend on the higher proportion of patients using atypical drugs, hence on a possible bias attributable to that these patients more probably switched to another atypical drug, which could not have been detected because we limited the attention on classes of drugs rather than on molecules. Nevertheless, the difference of persistence between the two classes of drugs appeared relevant. The approach we used to measure persistence in this study is not commonly applied and not comparable to those adopted in other studies conducted in this sector, where the observation was restricted to a limited number of drugs used (e.g., one out of 4 drugs in the CATIE randomized controlled study [19], one out of 7 drugs in the naturalistic study by Guo et al. [57]). However, because our study aimed at obtaining a real-world picture of our target population, we did not impose any criteria in regard to the treatment followed during the observational period. As a consequence, we enrolled patients that were using different combinations of many different antipsychotic drugs (18 in total), obtaining information that can actually be considered new for the community. Although not common, the approach of measuring persistence on classes of drugs can be useful to show the natural complexity of a treatment pattern, similarly to other areas, e.g., hypertension [58]. However, because we did not observe persistence between molecules, or even dosages, within the same class of drugs, we obtained results that should be considered conservative. Nevertheless, our results already show how complex is the antipsychotic treatment even considering only classes of drugs, and suggest a much higher complexity that can be relevant for the treatment decisions and related consequences. Fifth, although we observed interesting and promising trends in a prospective1-year observation, this time horizon is however too short to know the long term consequences related to the strategy adopted in schizophrenic patients. Finally, some criticisms could arise with regard to the approach used to estimate direct and indirect costs. Regarding direct costs, we did not estimate non-medical costs (e.g. costs of transportation, housekeeping): we chose this approach because we considered the informative gain attributable to these costs, which we expected not to be relevant if compared to the other costs, not enough to justify the additional cognitive burden that would be caused by requesting more details from the patients. Indirect costs were not monetized: we chose this approach because of the high risk of underestimating indirect costs in a population where many patients are still students, many others are idle or have an unpaid job. We consider it more accurate and informative to describe and provide a picture of the study sample according to the patient’s educational and working status and to estimate the number of days that they and their caregivers miss work, school, or the possibility to do their usual activities.

As in the other studies focusing on costs e.g., [10,59], our estimates are not totally applicable to other health care systems, because of the unavoidable country specificity of some data (e.g., unit costs) and methodologies used to conduct the study and perform the analyses. To expand applicability of our results, among the results we specify the mean consumption of specific categories of resources, which could be multiplied by different unit costs that are applicable in other health care systems. In any case, by keeping in mind the possible differences and adjustments to be made between the different healthcare sectors, these estimates remain valid to allow the community to understand the type and amount of the implications that are related to the management of subjects with the condition under study.

## Conclusion

In conclusion, this study provides a real-world complete picture of persistence, compliance, healthcare costs, loss of productivity, health related quality of life and clinical outcomes in young schizophrenia patients treated with the several options available. Our results suggest how tailoring the treatment strategy according to the complex and specific patient needs is necessary to gain benefits and to make allocation of resources more efficient. Finally, this study can also provide information on the most relevant items to be considered when conducting cost-effectiveness studies comparing specific alternatives for the treatment of target patients.

## Competing interests

This study was financially supported with an unrestricted grant received from Janssen-Cilag Italy s.p.a.. Claudio Mencacci received research support from Eli Lilly Italy s.p.a. and Lundbeck Italy S.p.A.; Fabiana L Lopes and Maria G Giustra were employees of Janssen-Cilag Italy S.p.A.; Luciana Scalone, Ferrannini Luigi, Elvezio Pirfo, Patrizia Berto, Miriam CJM Sturkenboom, Paolo A Cortesi and Lorenzo G Mantovani have no conflict of interest to declare.

## Authors’ contributions

CM, FL, EP, MCJMS, and LGM contributed to the design and overall conduct of the study. LS and PAC drafted the manuscript. FLL, MGG, CM, FL, EP, MCJMS and LGM reviewed the manuscript draft critically and made important contributions to its revision. LS and PAC contributed to the coding and analysis of data. CM, FL, EP, FLL and MGG contributed to the acquisition of data and in terms of the medical knowledge required for this study. LGM, LS, MCJMS and PAC contributed in terms of the health economics and patient reported outcomes knowledge for this study. All authors contributed to the interpretation of results. All authors read and approved the final manuscript.

## Pre-publication history

The pre-publication history for this paper can be accessed here:

http://www.biomedcentral.com/1471-244X/13/98/prepub
